# Analysis of apoptosis related genes in nurses exposed to anti-neoplastic drugs

**DOI:** 10.1186/s40360-019-0372-0

**Published:** 2019-12-02

**Authors:** Maral Ramazani, Razieh Pourahmad Jaktaji, Farshad H. Shirazi, Maria Tavakoli-Ardakani, Ahmad Salimi, Jalal Pourahmad

**Affiliations:** 1 Department of Pharmacology and Toxicology, Faculty of Pharmacy, Shahid Beheshti University of Medical Sciences, Tehran, Iran; 2 Department of Genetics, Faculty of Sciences, Shahr-e-Kord University, Shahr-e-Kord, Iran; 3 Department of Clinical Pharmacy, Faculty of Pharmacy, Shahid Beheshti University of Medical Sciences, Tehran, Iran; 4 Department of Pharmacology and Toxicology, School of Pharmacy, Ardabil University of Medical Sciences, Ardabil, Iran; 5 Pharmaceutical Sciences Research Center, Shahid Beheshti University of Medical Sciences, Tehran, Iran

**Keywords:** Nurse, Apoptosis, Gene analysis, Mitochondria, Anti-neoplastic drugs

## Abstract

**Background:**

Anti-neoplastic agents are widely used in the treatment of cancer and some non-neoplastic diseases. These drugs have been proved to be carcinogens, teratogens, and mutagens. Concern exists regarding the possible dangers of the staff handling anti-cancer drugs. The long-term exposure of nurses to anti-neoplastic drugs is still a controversial issue. The purpose of this study was to monitor cellular toxicity parameters and gene expression in nurses who work in chemotherapy wards and compare them to nurses who work in other wards.

**Methods:**

To analyze the apoptosis-related genes overexpression and cytotoxicity effects, peripheral blood lymphocytes obtained from oncology nurses and the control group.

**The results:**

Significant alterations in four analyzed apoptosis-related genes were observed in oncology nurses. In most individual samples being excavated, *Bcl-2* overexpression is superior to that of *Bax*. Prominent *P53* and *Hif-1α* up-regulation were observed in oncology nurses. Moreover, all cytotoxicity parameters (cell viability, ROS formation, MMP collapse, Lysosomal membrane damage, Lipid peroxidation, Caspase 3 activity and Apoptosis phenotype) in exposed oncology nurses were significantly (*p* < 0.001) higher than those of unexposed control nurses. Up-regulation of three analyzed apoptosis-related genes were observed in nurses occupationally exposed to anti-cancer drugs.

**Conclusion:**

Our data show that oxidative stress and mitochondrial toxicity induced by anti-neoplastic drugs lead to overexpression of apoptosis-related genes in oncology nurses.

## Background

Chemotherapy is an important method for cancer treatment that is used for anti-cancer drugs to cure patients. Anti-neoplastic drugs are extremely active biological compounds and their actions are non-selective [1]. Previous studies showed the possible dangers there to the staff handling anti-cancer drugs [2, 3]. Mutagenic and carcinogenic effects of anti-cancer drugs were shown in 1969 by the International Agency for Research on Cancer [4, 5]. Mutagenesis, chromosomal damages, skin hyperpigmentation, abortion, premature birth, and abnormal pregnancies are shown in that people who are in contact with anti-neoplastic drugs [3]. Anticancer drugs can also produce reactive oxygen species (ROS) which purportedly leads to mutations and DNA damage. Overproduction of ROS can affect lipids, protein, and DNA of the cell and lead to destroying the structure and function of cells [6]. At low doses, ROS had been associated with induction of cell survival signaling pathways, whilst at high doses activate death signaling through apoptosis and necrosis [7]. Oxygen-free radicals activate the tumor suppressor protein p53 at lower doses causes DNA repair, cell cycle arrest, and senescence. Well, if damages were very intense, p53 can control apoptosis transcriptionally by up-regulating pro-apoptotic members such as Bax, Bid, Puma, Noxa and Apaf-1, DR-4, FasL, Fas and DR-5 and down-regulating pro-survival proteins such as IAPs, Bcl-XL, Bcl-2 and surviving [8]. In the cytosol, P53 can translocate to mitochondria and interact straightly with Mcl-1, Bcl-XL and Bcl-2 and the pro-apoptotic proteins Bak and Bax, which lead to mitochondrial membrane potential (MMP) collapse, the release of pro-apoptotic factors and apoptosis. Overexpression of the pro-apoptotic proteins can lead to increased mitochondrial membrane collapse and release of cytochrome c, which finally can lead to ectopic apoptosis in lymphocytes [8, 9].

It is well known that oncology nurses are subject to many antineoplastic drugs. Nurses are exposed to anticancer drugs during preparation and administration. Exposure to antineoplastic drugs can occur mainly to hands and occasionally to other body parts or through contact with patients treated with anti-cancer drugs via their clothing and excreta [10, 11]. There several studies about the evaluation of anti-cancer drug apoptosis in lymphocytes of nurses [12–14]. Often these studies are focused on identifying a new diagnostic marker for nurses exposed to anti-cancer drugs in workplaces. We recently studied mitochondrial and cytotoxicity parameters in lymphocytes and isolated mitochondria of oncology nurses. Our results showed that mitochondrial toxicity and cytotoxicity parameters in the exposed group were remarkably increased compared to the unexposed group. Also, genotoxic, cytotoxicity and apoptosis monitoring in nurses handling cytotoxic drugs indicated chromosomal abnormalities, oxidative stress and cell death in the peripheral blood lymphocytes [15]. The present study focused on the analysis of apoptosis-related genes, for this purpose, we monitored cellular toxicity parameters and gene expression in nurses who work in chemotherapy wards and compare it to nurses who work in other wards.

### Methods

#### Sample collection

All nurses that work in chemotherapy wards of Shahid Beheshti University hospitals were chosen by entering and exit criteria by physician test. The minimum working time in the chemotherapy ward was 6 months and 45 h per week. Demographic data such as age, sex, time exposure, smoking status, and alcohol drinking were collected. The sample size was determined based on the power and standard deviation of variables in previous studies by power and sample size program software (50 nurses for each group).

Blood samples were collected (about 15 ml) in tubes containing EDTA-K2 anticoagulant agent, nurses were fasting. Control nurses who work in other wards of hospitals were chosen by the same condition as exposure nurses. All experiments were performed at Shahid Beheshti University of Medical Sciences.

### Isolation of lymphocytes

The nurse’s lymphocytes were isolated using the Ficoll standard method. Diluted blood samples with normal saline were layered on 3 ml Ficoll-Paque PLUS, at 2500 rpm for 20 min and lymphocyte layer was centrifuged and collected, the obtained cells were suspended in erythrocyte lysis buffer (10 mM NaHCO3, 1 mM EDTA, 150 mM NH4Cl, pH 7.4), and incubated at 37 °C for 5 min. The PBS was immediately added, and the lymphocytes were centrifuged at 1500 *g* and 20 °C, for 10 min. The supernatant was discarded, and the lymphocytes were washed twice with RPMI1640. The lymphocytes were resuspended in RPMI1640 medium with 10% FBS and L-glutamine, using trypan blue exclusion dye were counted. The cell viability was over 95% and lymphocytes were retained in a humidified atmosphere with 5% CO2 at 37 °C. The cell concentration used in each test was 10 × 106 cells/ml [15].

### MTT assay

The viability assessment was measured by the MTT method. The absorption of formazan dyes was read at 570 nm wavelength by an ELISA plate reader. All data were confirmed by using replication for at least three identical experiments [16].

### Annexin V/Propidium iodide staining

Apoptosis and necrosis were determined by using the apoptosis detection kit (K101 BioVision, CA, USA). In summary, lymphocytes were resuspended in 500 μl binding buffer and then FITC-conjugated Annexin V and propidium iodide (PI) were added and after 5-min incubation, samples were monitored by flow cytometer (Becton–Dickinson, USA) [17].

### Caspase 3 activity assay

Activation of caspase 3 was determined in lymphocytes by Sigma’s caspase-3 assay kit (CASP-3-C). The hydrolysis of a substrate peptide, Ac-DEVD-PNA, by caspase-3 is the base of this colorimetric assay. The concentration of p-nitroaniline (μM) released from the Ac-DEVD-PNA is computed from absorbance values obtained at 405 nm wavelength by an ELISA plate reader. Defined, p-nitroaniline concentrations were used for the preparation of a calibration curve [17].

### ROS detection

To measure the rate of lymphocytes ROS formation, dichlorofluoresceindiacetate (DCFH-DA, 1.6 μM) was added to the lymphocytes. The fluorescence intensity of dichlorofluorescein (DCF) was detected at excitation and emission wavelengths 490 nm and 525 nm, respectively by using a fluorescence spectrophotometer [16].

### Lipid peroxidation assessment

Lipid peroxidation detection in lymphocytes was performed by measuring the amount of malondialdehyde (MDA) during the disintegration of lipid hydroperoxides by monitoring the absorbance at 532 nm in a spectrophotometer [16].

### Lysosomal damage assay

Lysosomal damages in lymphocytes were measured by acridine orange dye. Acridine orange that was remaining in the cell suspension was measured at 495 nm excitation and 530 nm emission wavelengths by a fluorescence spectrophotometer [16].

### MMP assay

Mitochondrial uptake of Rhodamine 123 dyes (1.5 μM), has been applied for estimating MMP collapse. The amount of Rhodamine 123 remaining in the incubation media was measured at 470 nm excitation and 540 nm emission wavelengths by a fluorescence spectrophotometer [16].

### RT-qPCR analysis

The expression of six genes (*Bcl-2, Bax, P53, TopIIa, TopIIb, and Hif-1a*) was determined using RT-qPCR, primer sequences for genes shown in Table 1. Total RNA was extracted from lymphocyte samples using the RNeasy mini kit (including RNase free DNaseI) (Qiagen, Germany). Using electrophoresis in 1.2 agarose gel all extracts were qualitatively evaluated and spectrophotometrically quantitated (Ultrospec 1100, Amersham Pharmacia Biotech). 1 μg of total RNA was exposed to cDNA synthesis. First-strand cDNA was produced using the RevertAid Reverse Transcriptase kit (Yekta Tajhiz Co., Iran). Primers for amplification of these genes were listed in Table 1. The real-time quantitative RT-PCR analysis and amplification of cDNA were performed using a Rotor-Gene 6000 thermocycler (Corbett Research, Australia) using an SYBR Green kit (Yekta Tajhiz Co., Iran). The thermal condition was as follows: Pre-denaturation at 95 °C for 5 min, denaturation at 95 °C for 10 s, annealing at 60 °C for 20 s, extension at 70 °C for 20 s, 40 cycles in total. *β-actin* was used as a housekeeping gene to normalize the cDNA variation. Relative quantification was made using the Pffafl method (16). Each experiment was repeated three times [18].

**Table 1:** Primer sequences for six genes which chosen for gene expression analysis

Gene	Size of base pairs	Sequence of primer 5′ 3′
*Bax*	155	F: CCCGAGAGGTCTTTTTCCGAG R: CCAGCCCATGATGGTTCTGAT
*Bcl-2*	89	F: GGTGGGGTCATGTGTGTGTGG R: CGGTTCAGGTACTCAGTCATCC
*P53*	181	F: GTCTGGGCTTCTTGCATT R: GTCATGTGCTGTGACTGC
*ACTB*	138	F: AACGGTGAAGGTGACAGCAGTCG R: GGCAAGGGACTTCCTGTAACAACG
*Hif-1a*	164	F: ACAGCCTCAGGAAACAGAGCAGG R: CGCTTTCTCTGAGCATTTGCAAAGC
*TopoIIa*	175	F: GAATCAGATAGGAGCAGTGACG R: GTGGACTAGCATCTGATGGGAC
*TopoIIb*	182	F: GCACTGACCTGGGTGAACAA R: ACCCACATGAACTGCGTCAA

### Statistical analysis

The unpaired T- test was used to compare the results between two nurses’ groups using GraphPad Prism 7 software, with *P* < 0.05 being significant. For RT-qPCR, relative quantification was made using the Pffafl method and another analysis was performed by the Two-way ANOVA test and Bonferroni post-test. Based on pffafle analysis, a ratio above 2 is considered as Overexpression and below 0.5 as Low Expression.

## Results

### Demographic data

**Fig. 1:**
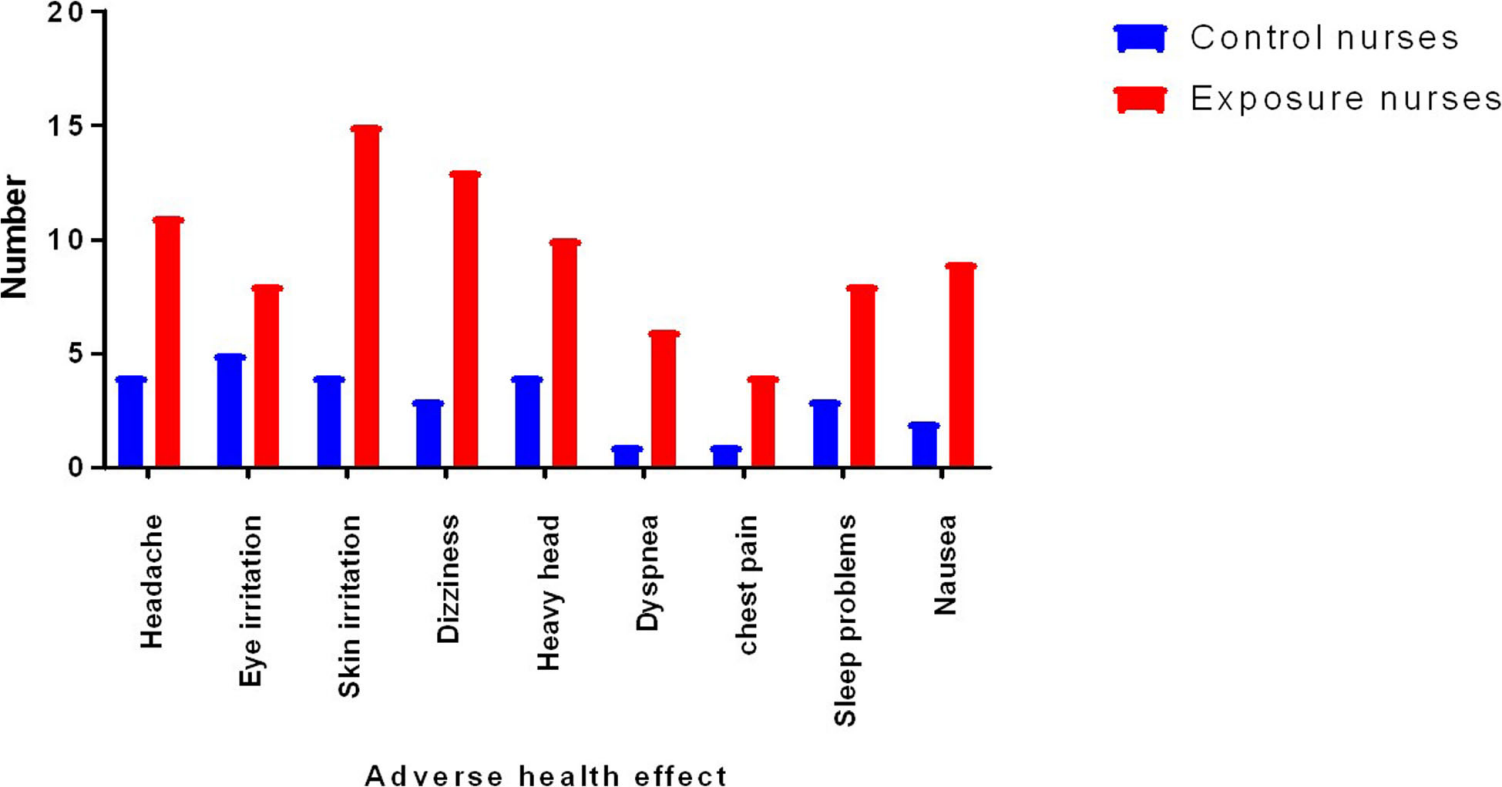
The number of symptoms observed in exposed and unexposed nurses to antineoplastic drugs. Two groups analyzed by the chi-square test. All of the above-mentioned symptoms were obviously higher in nurses in the exposed group compared to the unexposed group

Important demographic data were shown in Table 2. 83.33% of nurses were women and 16.67% were men in both groups. The average age of oncology nurses was 29.27 years and in the control group were 28.69 years. Control nurses never work in the oncology ward and an average of times of exposure to oncology nurses was 27.43 months. A series of general clinical symptoms, including a headache, eye irritation, skin irritation, dizziness, dyspnea, chest pain, sleep problems, and nausea were evaluated in nurses in both control and exposed groups. All of the above-mentioned symptoms were higher in nurses in the exposed group, but most of these nurses complained of skin irritation, dizziness, a headache, and heavy headache (Fig. 1).

**Table 2:** The demographic characteristics of study nurses

	Control nurses	Oncology nurses
Male	16.67%	16.67%
Female	83.33%	83.33%
Smoking	0	0
Alcohol drinking	0	0
Average of age (year)	28.69 ± 4.008	29.27 ± 3.693
Average time of work in oncology ward (month)	0	25.43 ± 27.73
Antioxidant therapy intake	> 1%	> 1%
Use of personal protection tools	99%	99%
Interference in preparation drug stage	100%	0
Pregnance nurses (number)	0	2 ^*^

*pregnancy nurses removed from this study

### Viability assessment

**Fig. 2:**
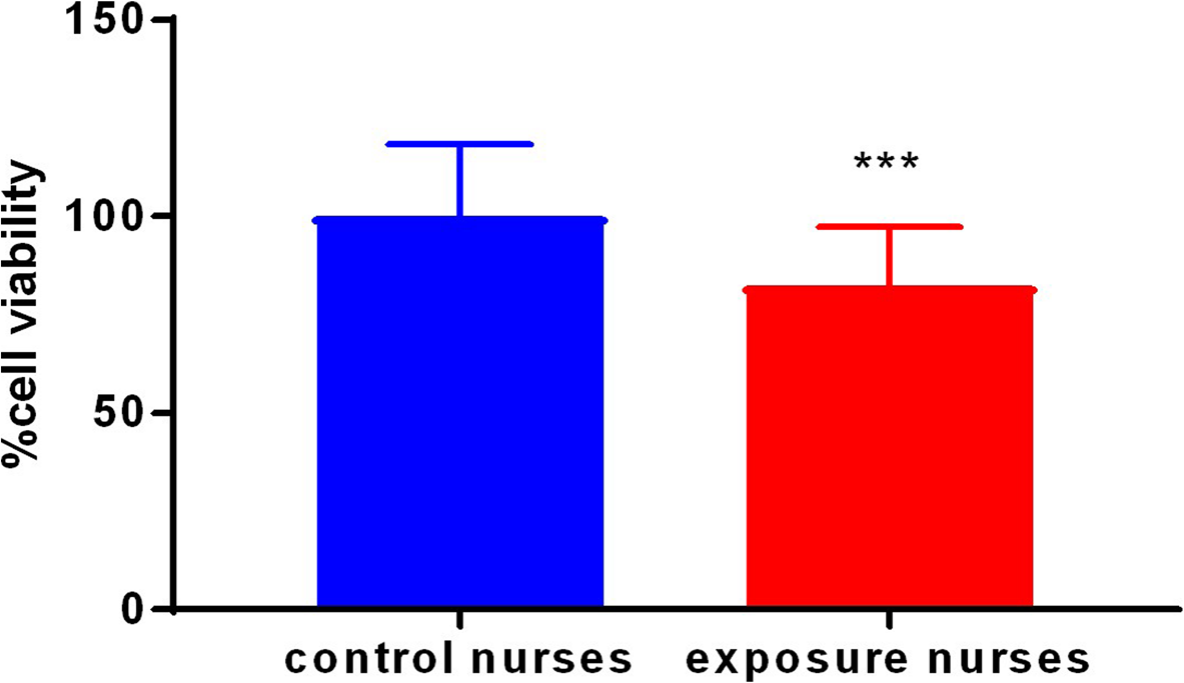
The viability of lymphocytes isolated in exposed and control nurses, cell viability were significantly higher in nurses in the exposed group compared to the unexposed group. Values (mean ± SD) are from three independent experiments (*n* = 3). ***p < 0. 001. Results are expressed as means ± SD, *n* = 50

According to Fig. 2, the lymphocyte viability of oncology nurses was significantly lower than those of control nurses (*p* < 0.001).

### Apoptosis assay

**Fig. 3:**
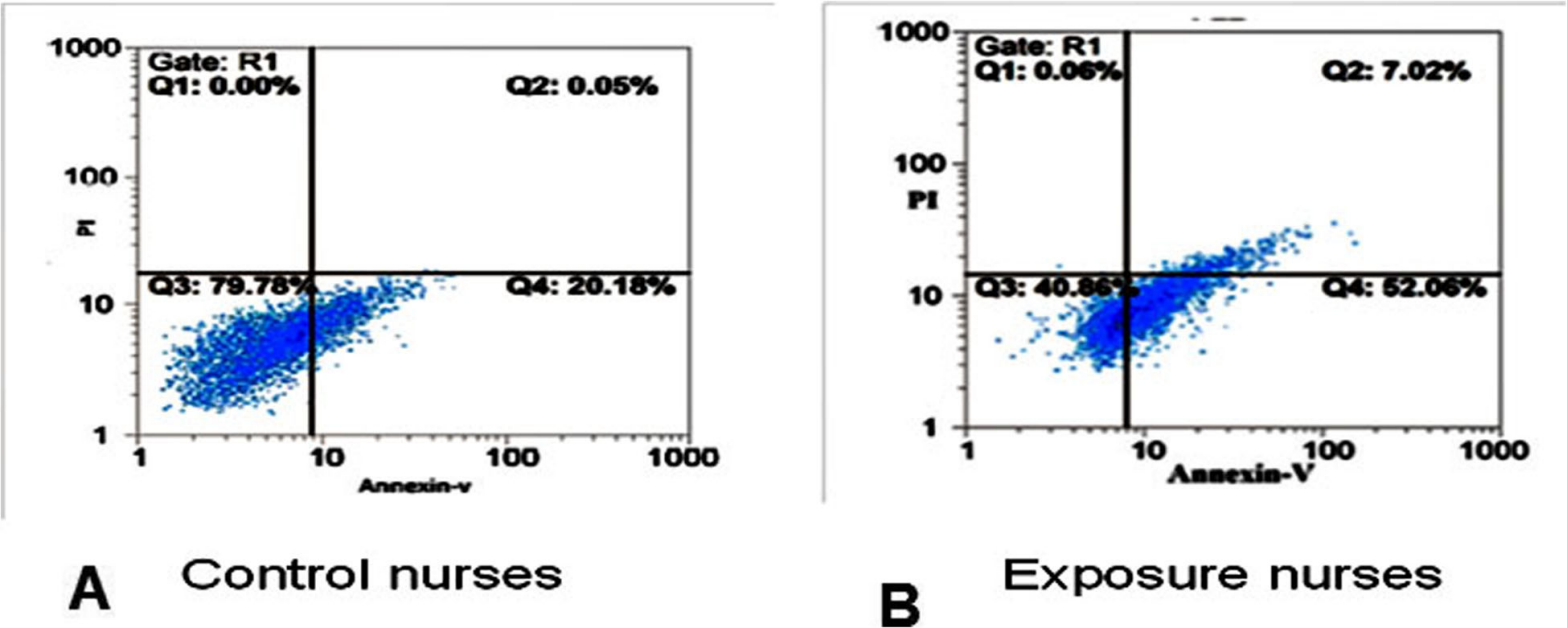
Apoptosis assay in exposed and unexposed nurses. **a** Unexposed nurses; (**b**) exposed nurses. Apoptosis was measured by the annexin V assay using flow cytometry. The assay gives information about the number of live cells (AV−/PI-), apoptosis cells (AV+/PI-), necrotic cells (AV−/PI+) and secondary necrosis cells (AV+/PI+)

Using cell flow cytometry and AnnexinV-PI staining, cell status was determined for apoptosis and necrosis in control and exposed groups. According to the data, lymphocytes which were at apoptosis state in chemotherapy nurses were more than in control nurses (*P* < 0.001) which are shown in Fig. 3. Quadrant 1 includes necrosis cells (Annexin V−/PI+), Quadrant 2 includes cells under secondary necrosis or late apoptosis (Annexin V+/PI+), Quadrant 3 includes live cells (Annexin V−/PI-) and Quadrant 4 includes cells under early apoptosis (Annexin V+/PI-).

### Activation of Caspase 3

**Fig. 4:**
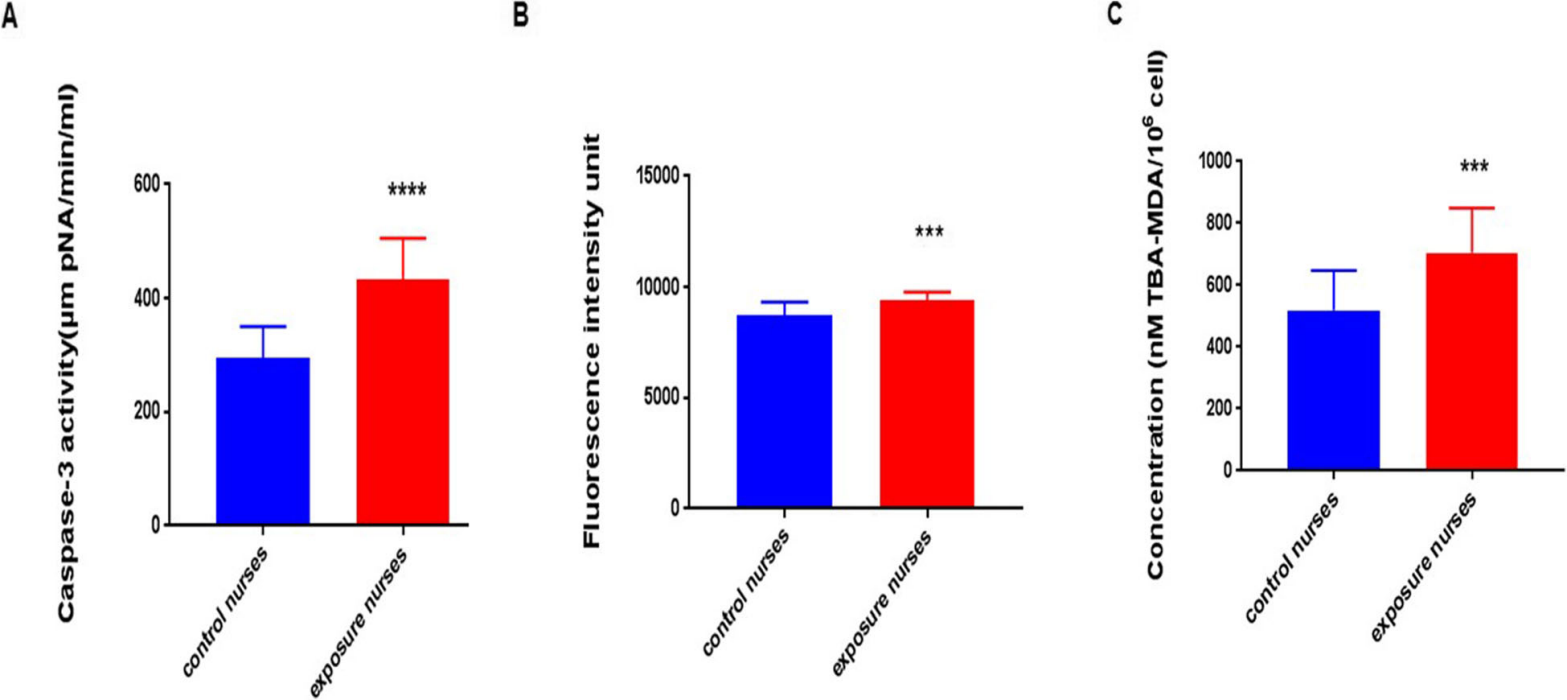
Caspase-3 activity (**a**). ROS formation was measured (**b**). Malondialdehyde amounts formed during the decomposition of lipid hydroperoxides (**c**). All of them was significantly higher in nurses in the exposed group compared to the unexposed group. ***p < 0.001 ****p < 0. 0001. Results are expressed as means ± SD

The activity of caspase 3 in cytoplasm starts a process that leads to apoptosis in cells. This activation was measured by a substrate of caspase 3. Figure 4a shows that the activity of caspase 3 in lymphocytes isolated from oncology nurses has been signed (*p* < 0.001) higher than that of control nurses.

### Measurement of ROS formation

Disruption of the balance between cellular ROS formation and endogenous antioxidants can start oxidative stress in cells. Our results show that the amounts of ROS formation in lymphocytes of oncology nurses were significantly (p < 0.001) higher than those of control nurses (Fig. 4b).

### Lipid peroxidation in isolated lymphocytes

Lipid peroxidation was determined by measuring the amount of malondialdehyde (MDA) in isolated lymphocytes. As shown in Fig. 4c, lymphocytes of oncology nurses have significantly (*p* < 0.001) higher than MDA compared to those of control nurses.

***Lysosomal membrane integrity.***

**Fig 5:**
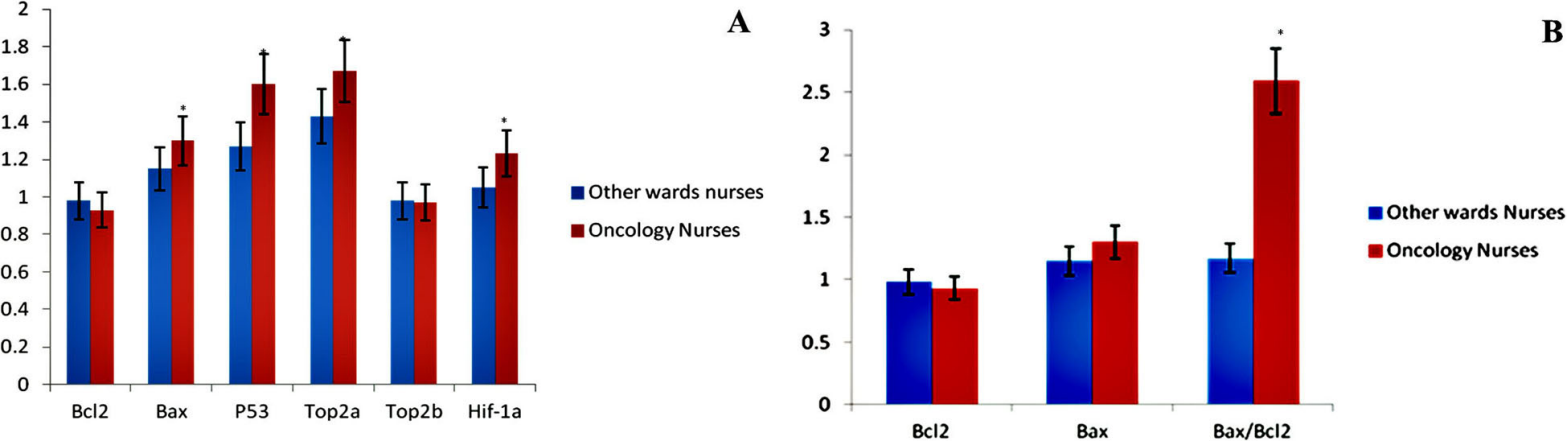
Lysosomal membrane damage (**a**). MMP collapse. **b**. Two parameters were significantly higher in nurses in the exposed group compared to the unexposed group Each bar represents mean ± SD (n = 50). **** *p* < 0.0001 significant differences compared to the unexposed group

Lysosomal integrity was determined by Acridine Orange fluorescent dye redistribution in lymphocytes isolated from the nurse’s blood. The results of the lysosomal integrity test show that nurses who work in the oncology ward have higher lysosomal membrane damage compared to those of control nurses (Fig. 5a) (*p* < 0.001).

### Mitochondrial membrane potential collapse

To understand changes in mitochondrial membrane potential, we used Rhodamine 123 dye. When the fluorescence intensity related to Rhodamine 123 release from mitochondria into the cytosol, is high, it means that the loss of mitochondrial membrane potential is also high. Our results showed that lymphocytes of oncology nurses demonstrate more decline in mitochondrial membrane potential rather than those of control nurses (Fig. 5b) (*p* < 0.001).

### Determination of Bax, Bcl-2, P53, Hif-1a, topoisomerase-IIa and topoisomerase-IIb level

**Fig 6:**
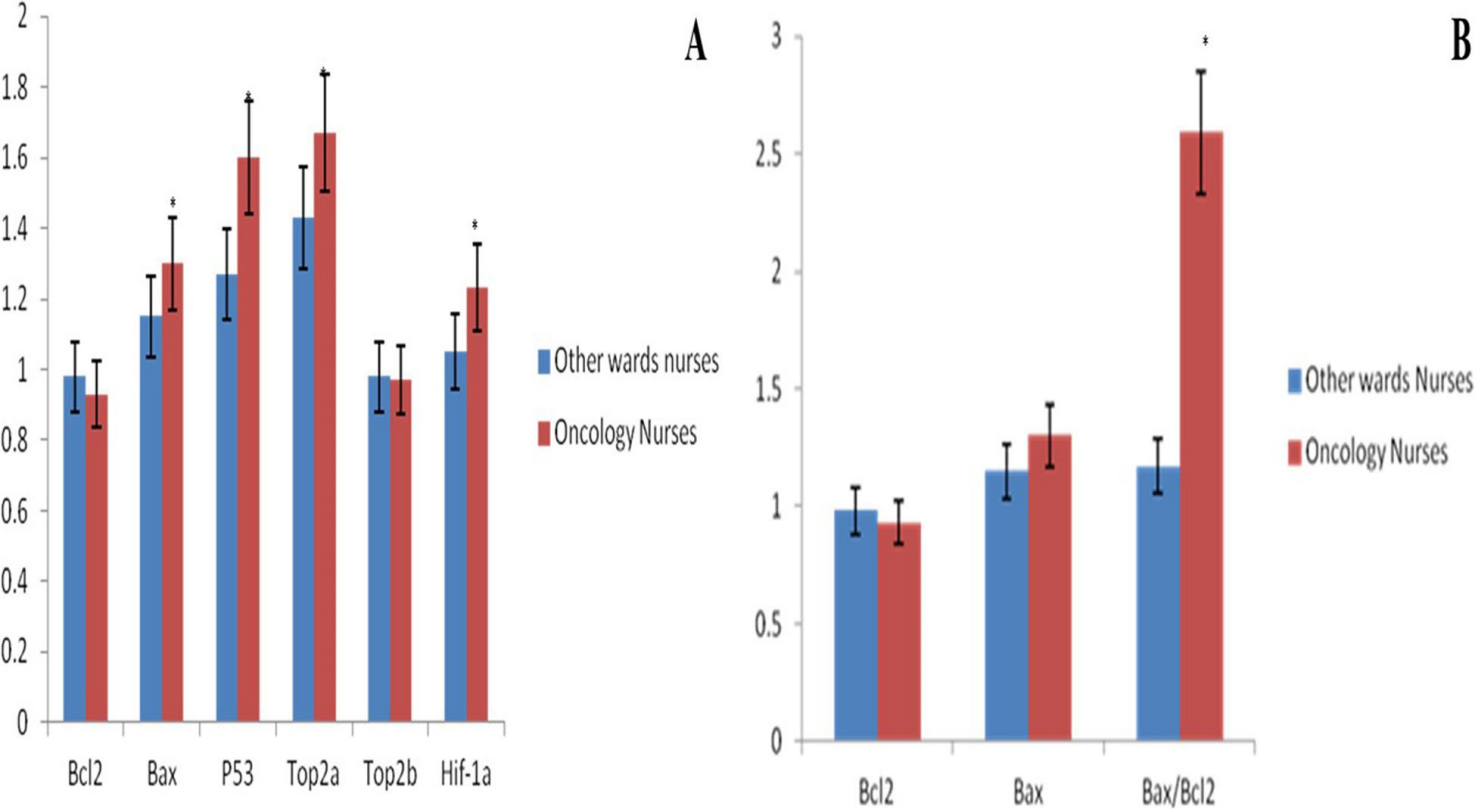
The relative expression of Bcl-2, Bax, P53, Top2α, Top2β, and Hif-1a (**a**) and relative expression of Bcl-2, Bax and Bax/Bcl-2 ratio (**b**) * indicated the significant difference (*p* < 0.01) between exposed with unexposed nurses. Results are expressed as means ± SD (*n* = 50)

Relative expression levels of six genes with central roles in the regulation of apoptosis, chromosome segregation, transcription, DNA repair, and glucose metabolism were analyzed by RT-qPCR in two groups of nurses (oncology and controls). Data analysis of 50 exposed nurses demonstrated reduced *Bcl-2* expression (significantly reduced expression value, less than 0.5) in 20 cases (40%) and increased *Bcl-2* expression in 15 cases (30%) (not significantly overexpression, a value less than 2) and approximately equal expression of *Bcl-2* (normal expression) in 15 remaining cases (30%). The expression of this gene was nearly the same in cases in the control group. A comparison of the average expression of *Bcl-2* in two groups did not show a significant difference between them (*p* > 0.05). Whereas the expression of *Bax* increased significantly (*P* < 0.05) in 20 cases (40%) and the remaining was nearly the same in the rest of exposed nurses. Cases in the control group showed normal similar expression. A comparison of the average expression of *Bax* in two groups did not show a significant difference between them (p > 0.05). The real-time PCR data demonstrated increased expression of *Bax*: *Bcl-2* ratio of 40% of exposed nurses as compared with control ones. The average expression of *Bax*: *Bcl-2* ratio of exposed nurses was significantly different from that in the control group (P < 0.05). *P53* was overexpressed in 20 cases (40%) in the exposed group. The average expression of *P53* in exposed nurses was significantly different from that in the control group. It is expected that the expression of wild type *Top2α* and *Top2β* decreases in chemotherapeutic resistant tumor cells. However, these two genes expressed similarly in two groups. The average expression of both genes in exposed nurses was not significantly different from that in the control group. It was shown that *Hif-1α* overexpresses in chemotherapeutic resistant tumor cells, especially in acute lymphoblastic leukemia (ALL) and acute myeloid leukemia (AML). Whereas *Hif-1α* neither overexpressed nor showed a significant difference in two groups (Fig. 6).

## Discussion

We previously reported the results of mitochondrial toxicity and oxidative stress parameters in nurses that have been occupationally exposed to antineoplastic drugs [15]. In the current study, we searched apoptosis and expression of apoptosis-related genes in the same set of the antineoplastic drug-exposed nurses. According to biochemical observations, the obtained results in our study showed that the mitochondrial toxicity and oxidative stress parameters in the lymphocytes of nurses in the oncology ward are higher than significantly compared with other nurses. We confirmed that apoptosis in antineoplastic drug-exposed nurses was higher than unexposed nurses with antineoplastic drugs (Fig. 3).

Bcl-2 family proteins have a key role in the regulation of cell death [19]. There two types of proteins in this family, including the pro-survival subfamily like Bcl-2 band the pro-apoptotic subfamily such as Bax [20]. Anti-apoptotic *Bcl-2* family members support the viability of cells by binding to multidomain pro-apoptotic members and inhibiting apoptotic activities of them. However, multidomain pro-apoptotic Bcl-2 family members attack the mitochondria and lead to mitochondrial outer membrane permeability and apoptosis [21]. In this study, we showed that exposed nurses with antineoplastic drugs have higher pro-apoptotic Bcl-2 proteins than unexposed nurses (Fig. 6). These data are correlated with the induction of apoptosis, cytotoxicity and mitochondrial membrane potential collapse. Cellular ROS formation can be reduced by Bcl-2. Antioxidant effects of Bcl-2 are shown by previously published studies. These studies suggested that the pro-survival Bcl-2 family decrease glutathione depletion in cells [22, 23]. We observed a higher level of ROS formation in the exposed nurses compared with unexposed nurses. The obtained results suggested that a decrease in pro-survival Bcl-2 is effectively in ROS content in exposed nurses. Deactivation of the anti-apoptotic Bcl-2 family members leads to activation of the pro-apoptotic Bcl-2 such as Bak and Bax, these occurrences are thought to happen in parallel, finally resulting in cytochrome c release through MMP collapse, activation of caspases and apoptosis [21, 24]. Our results showed that an increase in *Bax/Bcl-2* is accompanied by an MMP collapse in mitochondria, activation of caspase 3 and apoptosis in exposed nurses (Figs. 3, 4A and 5B).

Hif-1*α* is remarked as the master transcriptional controller of development and cellular reply to hypoxia. The overexpression and dysregulation of Hif-1*α* by genetic alternations or hypoxia have been involved in the biology of cancer, specifically in areas of energy metabolism, angiogenesis and vascularization, tumor invasion and cell survival [25]. A recent study showed that Hif-1*α* is recruited to mitochondria in answer to oxidative stress and safeguards against oxidative stress-induced apoptosis. In mitochondria, Hif-1*α* decreases ROS levels and reverses mitochondrial damage [26]. Also, previous studies showed that HIF-1*α* decreases ROS formation via multiple pathways and reverses mitochondrial damage [27]. Our results here suggest that probably overexpression of *Hif-1α* in exposed nurses is for inhibition of mitochondrial and oxidative damage induced by exposing antineoplastic drugs.

Tumor suppressor P53 is a transcription factor that controls cell growth [28]. New investigations showed that P53 can lead to changing from normal to an abnormal condition under different stress conditions in mitochondria. When stress stimuli occur, like exposure to antineoplastic in our study, ROS promote P53 to repair dysfunctional mitochondria to finish mitochondrial degradation [29]. If damage or stress is irreversible, P53 will translocate to mitochondria, leading into necrosis or apoptosis [30].

Topoisomerase II is a type II DNA topoisomerase that has been reported to be expressed in all mammalian cells, but is widely expressed in cells that are differentiating for achieving post-mathisophytic status. Topoisomerase II plays an important role in various biological areas such as transcription, DNA repair, neurodegeneration, aging, HIV infection and cancer [31–35]. Exposure to anti-cancer drugs, especially those targeting DNA topoisomerases may change the expression pattern of *Top2α* and *Top2β* genes [36]. We did not find a significant difference in expression of these genes in two groups (*P* > 0.05) and both groups showed normal expression. Additionally, exposure to these drugs may cause resistance against them. *Hif-1α* can be used as a biomarker that shows resistance as its expression enhances in resistant cells [37]. Neither of the groups showed overexpression of this gene and a significant difference in the level of expression (*P* < 0.05).

We couldn’t detect all chemotherapy drugs concentration in the blood of nurses because the number of drugs that are in use in chemotherapy wards of our country Iran are more than 40 different drugs and measurement of their exact concentration in the blood sample is not possible at the same time. Besides, these drugs have synergistic and antagonistic effects on each other. Therefore, the presence or absence of a single drug in blood at the time of measurement is not a reason for the absence or present of adverse effects.

## Conclusions

In summary, the results of the current study showed that oxidative stress and mitochondrial toxicity induced by antineoplastic drugs lead to overexpression of apoptosis-related genes in human lymphocytes.

## Data Availability

All data and materials related to the study can be obtained in the Materials and Methods Section.

## Abbreviations

DCF: Dichlorofluorescein
DCFH-DA: Dichlorofluorescein Diacetate
IARC: International Agency for Research on Cancer
MDA: Malondialdehyde
MMP: Mitochondrial Membrane Potential
ROS: Reactive Oxygen Species
